# PARAFFIN: A software tool for Pathology Report Automated Feedback for Improved Education of anatomic pathology trainees

**DOI:** 10.1016/j.jpi.2025.100424

**Published:** 2025-02-13

**Authors:** Clarissa E. Jordan, Justin E. Juskewitch, Andrew P. Norgan

**Affiliations:** Department of Laboratory Medicine and Pathology, Mayo Clinic, Rochester, MN, USA

**Keywords:** Software, Automation, R, Pathology report, Feedback, Graduate medical education

## Abstract

**Background:**

Feedback on the diagnosis and reporting of pathology findings is essential to the training of residents and fellows, but time constraints and other factors can make it difficult to ensure learners are made aware of the outcome of all cases in which they participated. Many trainees attempt to keep track of their cases and later look up final pathology reports in the laboratory information system (LIS); however, this manual and time-consuming process is prone to error and may prevent them from spending time reviewing and learning from these reports.

**Methods:**

To address this, we developed a software solution, (**Pa**thology **R**eport **A**utomated **F**eedback **f**or **I**mproved Educatio**n**; “PARAFFIN”), which provides pathology trainees with a weekly email digest containing an attached case log with the date, accession sequence, attending pathologist initials, and final diagnosis text for each case in which they participated. PARAFFIN is implemented as two R scripts running on a Posit Connect server: a data extraction script, which accesses an interactive report from our enterprise analytics SQL server, and a reporting script, which performs recipient-specific filtering and emails the trainee with their personalized case log attached as .txt and .csv files. After implementation, pathology trainees were surveyed about PARAFFIN's impact on report collection and case feedback.

**Results:**

Of the total 51 pathology trainees who were receiving PARAFFIN digests at the long-term follow-up timepoint, 20 responded to our survey. 90% (18 of 20) of respondents report that PARAFFIN allows them to spend more time reviewing the content of final anatomic pathology reports, rather than collecting reports. Trainees report utilizing PARAFFIN for feedback on multiple aspects of pathology reporting, with final diagnosis, wording/style of final diagnostic line, and diagnostic comment being most frequently used.

**Conclusions:**

Our automated case feedback solution provides trainees with a record of final pathology reports for cases in which they participated, which allows trainees to spend more time reviewing reports for feedback rather than manually collecting them from the LIS.

## Introduction

### The importance of feedback in trainee education

Feedback is an essential component of learning for pathology trainees, but even in the most organized of environments, it can be difficult to ensure learners are consistently informed of the final outcome of cases in which they have been involved. As the work product of an anatomic pathologist is frequently a written report, examination of finalized reports is a valuable tool for trainees in establishing not only diagnostic accuracy but also knowledge of the nuanced conventions that govern the communication between pathologist and clinicians in many specialty areas. One-on-one teaching at the microscope (or digital display) during the process of evaluating a case (“sign-out”) plays a vital role in the education of a pathology trainee, but often occurs at a time when all relevant information may not be available. For example, the results of immunohistochemical stains or other ancillary studies (which will impact the diagnosis or other parts of the pathology report) may become available days after initial sign-out with the trainee, and time constraints often cause attending pathologists to modify and/or finalize cases when trainees are not available to participate. Ideally, such modifications are discussed with the trainee, but scheduling and other logistical issues sometimes prevent this important follow-up.

Previous studies at other institutions have noted that challenges in providing feedback to trainees are multifaceted, including social (related to the attending giving the feedback or the trainee receiving the feedback), as well as environmental factors.^1^ Increasing clinical demands, time constraints, and lack of an appropriate setting are consistently reported environmental factors affecting the delivery of timely feedback to trainees.^2^, ^3^, ^4^ For example, a study conducted at a radiology training program (which operates under a similar paradigm to pathology training programs, with attendings editing reports initially written by trainees), notes that a high volume of cases, lack of time, and spatial separation can prevent attending radiologists from closing the feedback loop with trainees and discussing diagnostic and/or stylistic changes.^5^ At our institution, a survey of trainees identified several perceived barriers to trainee receipt of feedback on all cases (Table 1), including lack of a formal/standardized process for providing feedback, limited time available to attending pathologists to provide feedback, and logistical challenges trainees experienced in logging and following up independently on cases after sign-out.

**Table 1:** Potential causes for lack of feedback on final pathology reports.

*Causes for lack of attending-initiated feedback*
Lack of a formal/standardized process for providing trainees with feedback on final pathology reports
Lack of dedicated time for attendings to provide trainees with feedback on their final pathology reports for all cases which they participated in
Competing interests (e.g., the presence of other pending cases) leading to decreased amount of attending time available for review/feedback with trainee

*Causes for lack of trainee-initiated feedback*
Lack of a method for trainees to easily retrieve from the laboratory information system (LIS) the accession/record numbers for cases in which they participated (i.e., no way for trainees to search the LIS for their own name to see record numbers for cases in which they participated)
Lack of a method for trainees to easily retrieve from the LIS the final pathology reports for cases in which they participated
Lack of trainee time available to manually keep track of final pathology reports

*Systemic causes for lack of feedback*
High volume of cases (limits ability to log and track)
High complexity of a subset of cases (extends turnaround time of cases so that trainees are no longer able to be involved in the case when completed)
Inability to direct cases (slides and paperwork) back to trainee after sign-out
Trainee may rotate to a different service before ancillary studies required for the final diagnosis are available, making both attending- and trainee-initiated opportunities for feedback more difficult

### Existing paradigm

Through the laboratory information system (LIS), trainees are typically afforded access to the final diagnosis of any case in which they have participated, but utilizing this resource on a consistent basis for case follow-up can be impractical. Many trainees in our institution reported that in order to ensure awareness of final sign-out diagnoses on their cases, they maintained a log of cases seen and subsequently performed a manual audit of their cases in the LIS to access the final pathology report for each case “one-by-one”. This audit was reported to be both tedious and time-consuming, with additional copy–paste steps required if trainees wished to maintain a more permanent record of their cases. Many trainees felt that the process of obtaining final sign-out diagnoses for their cases was sufficiently time-consuming that it limited how much time they were able to spend reviewing and learning from reports. Additionally, cases were inevitably missed due to omissions in logging or the lack of time to perform report retrieval.

### Goals of the project

While many potential barriers to improving trainee access to case feedback would require organizational, policy, or behavioral changes (e.g., case volume, rotation scheduling, individual attending, or trainee learning behaviors), we determined that a particular barrier, the time required to access final sign-out diagnosis through the LIS, was amenable to a technical solution. Therefore, we developed a software solution (**Pa**thology **R**eport **A**utomated **F**eedback **f**or **I**mproved Educatio**n**; “PARAFFIN”) that provides all trainees a formatted case log with the final report for any case in which they participated, including anatomic/surgical pathology, cytopathology, hematopathology, and autopsy pathology cases. By removing any barriers to access, this tool ensures that trainees can fully integrate their case experiences, maintain a log of the diagnoses/cases they have experienced throughout residency and/or fellowship, and have access to the precise wording of reports in order to facilitate their growth in the vocabulary and communication conventions in pathology.

## Materials and methods

### Implementation of PARAFFIN

**Fig. 1:**
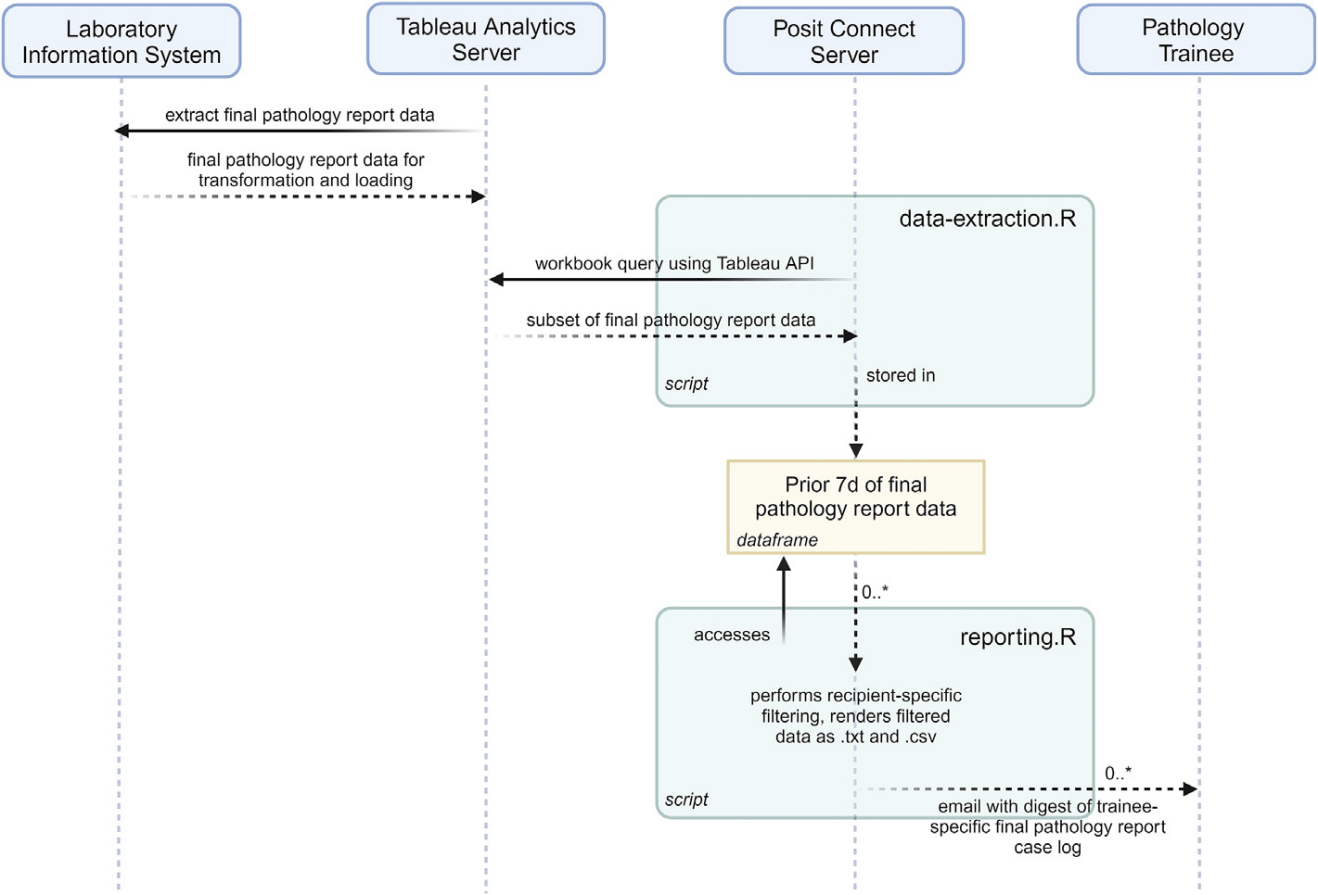
Sequence diagram of PARAFFIN implementation, demonstrating the interactions between the laboratory information system, enterprise analytic server (Tableau), Posit Connect server, and pathology trainee, as well as the main functions of the data extraction script and reporting script. The output of the reporting script is an email with a recipient-specific case log of final pathology reports that were signed out the previous week (attached as .txt and .csv files). Email digests are scheduled in Posit Connect to be sent every Saturday at 8:30 am. Created with BioRender.com.

We developed a scheduled reporting system that generates a weekly digest of case reports for each recipient and sends the report to the recipient through our enterprise email system. The technical implementation relies on two major components, an interactive report in our enterprise reporting system (Tableau, Inc.) and R^6^ scripts (data-extraction.R and reporting.R) running on an access-controlled Posit Connect server^7^ (Fig. 1). Tableau accesses an intermediate table generated daily by a scheduled extract-transform-load operation from the LIS (SCC SoftPath DX) to an analytics SQL server. The Tableau workbook has a view which allows filters of interest to be applied to the data (e.g., date, accession sequence, etc.). The ‘data-extraction’ R script scheduled to run on the enterprise Posit Connect servers utilizes the Tableau application programming interface (API) to query this view with appropriate filters to generate a table of last 7 days of sign-out activity; that query result is streamed to the Posit Connect server to populate an R dataframe. Importantly, this temporary table contains a column with IDs corresponding to trainees that participated in the case (in our institution, trainees are required to indicate their involvement in a case with a single button click that records their ID in a field within the LIS). Other data collected include the date the case was signed out, the accession sequence, the ID corresponding to the attending pathologist who signed out the case, and the full text of the pathology interpretation (up to 4000 characters due to Tableau character limits), including final diagnosis, morphologic description, ancillary studies, and synoptic report.

**Fig. 2:**
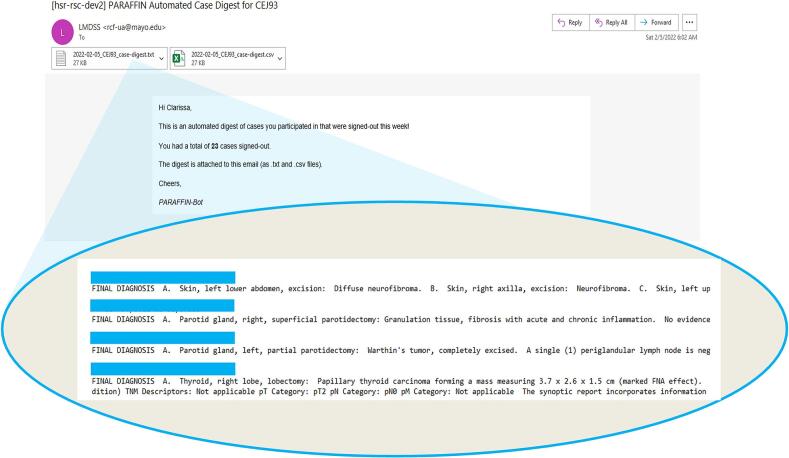
The body of the email summarizes the number of cases that were signed out in the previous week. The final anatomic pathology reports are attached as .txt and .csv files.

The “reporting” R script then parses the temporary table using a Parameterized Report template that generates a trainee-specific table for each trainee. This trainee-specific case log is rendered as .txt and .csv formatted files, and then emailed to the recipient using an email template prepared with the Blastula package^8^ (Fig. 2). Output is suppressed if the recipient did not participate in the interpretation of any cases that were signed out during the previous week. The weekly execution of this reporting script and delivery of its email output are automated using Posit Connect's built-in scheduling functionality. Recipients are scheduled to receive their email digest each Saturday at 8:30 am.

Future pathology trainees matriculating at our institution are added to the PARAFFIN system at the beginning of their training period and graduating pathology trainees leaving our institution are removed from the PARAFFIN system at the end of their training program.

### Budget

The budget for PARAFFIN is minimal, given that our implementation utilized available enterprise infrastructure and is comprised of only 150 lines of R code. The initial version was developed in approximately 8 hr (using some pre-existing code chunks). Maintenance of the PARAFFIN application (e.g., updating the recipient list and automated reporting) occurs on an annual basis and is conducted by the application developers (estimated <20 hr per academic year).

### Impact study

To evaluate the impact of PARAFFIN, a pre-intervention survey was distributed to trainees via REDCap survey and database software to capture baseline data, including participants' experience with feedback on reports and estimation of time spent gathering reports or creating case logs. A similar post-intervention survey was distributed after PARAFFIN implementation, and long-term re-measurement was conducted after 2 years. The percentages of trainees who received feedback on >75% of final anatomic pathology reports (as reported in the pre- and post-intervention surveys) were compared using the Chi-square test with Yates continuity correction; p-values <0.05 were considered significant in this study.

Study data were collected and managed using REDCap electronic data capture tools hosted at our institution.^9^^,^^10^ REDCap (Research Electronic Data Capture) is a secure, web-based software platform designed to support data capture for research studies, providing: (1) an intuitive interface for validated data capture; (2) audit trails for tracking data manipulation and export procedures; (3) automated export procedures for seamless data downloads to common statistical packages; and (4) procedures for data integration and interoperability with external sources.

This study was approved as an exempt study by the local Institutional Review Board.

## Results

A baseline survey was sent to all 43 pathology trainees at our institution, with 32 responding. The trainees surveyed were a mix of anatomic and clinical pathology residents, surgical pathology fellows, subspecialty anatomic pathology fellows, and hematopathology fellows, ranging in experience from post-graduate year 1–6. A follow-up survey after the implementation of PARAFFIN was sent approximately 5 months later to all 32 original pathology trainees who provided the baseline measurement. 31 trainees responded, of whom 27 received PARAFFIN digests (reasons for not receiving PARAFFIN digests include trainee participation in non-anatomic pathology rotations and improperly logging trainee participation in a case). A second follow-up survey was sent to all 51 pathology trainees at our institution approximately 2 years later, with 20 responding, all of whom reported receiving PARAFFIN digests. Although a subset of trainees may have participated in all three surveys, sub-analysis of this population was not performed due to respondent anonymity.

**Fig. 3:**
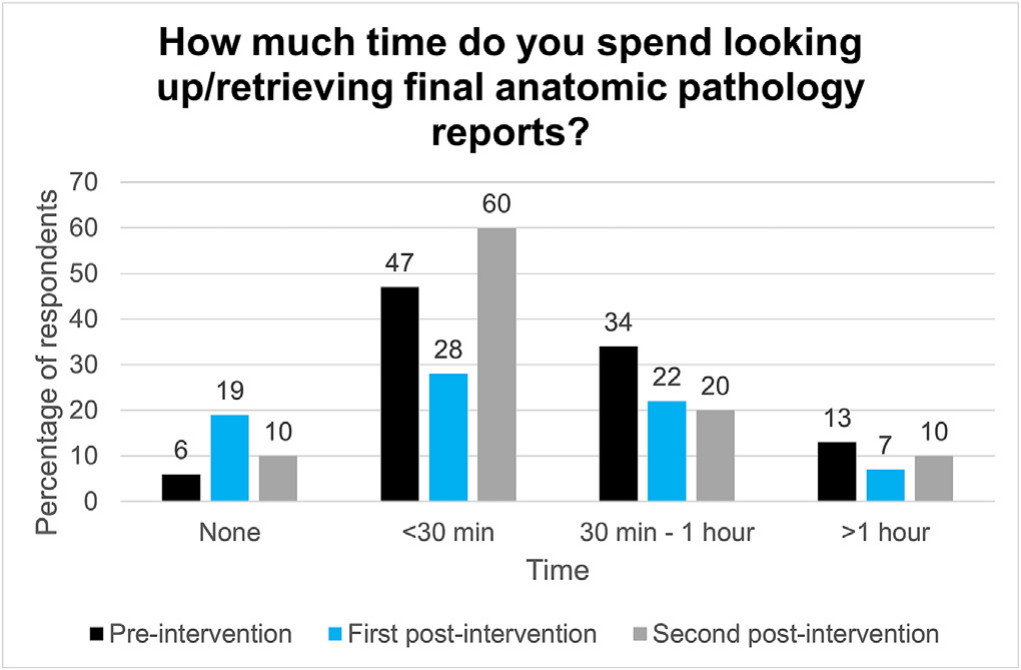
Survey results for the question “Over the course of an average week, how much time per day do you spend looking up/retrieving final anatomic pathology reports?”. Answer options were “none”, “<30 min”, “30 min–1 hr”, and “>1 hr”. (*n* = 32 respondents for the pre-intervention survey, *n* = 27 respondents for the first follow-up survey, and *n* = 20 respondents for the second follow-up survey).

Survey results demonstrate that PARAFFIN was successful in reducing the time burden of collecting final anatomic pathology reports for feedback and review. 47% (15 of 32) trainees reported spending at least 30 min per week looking up final anatomic pathology reports pre-intervention, which decreased to 37% (10 of 27) in the first post-intervention survey and subsequently 30% (6 of 20) in the second post-intervention survey (Fig. 3). We are uncertain of the reason for the increase in trainees who spent <30 min looking up final anatomic pathology reports during the second post-intervention survey period. One hypothesis is that trainees were more likely to look up any addenda pathology reports associated with their cases, after they began receiving PARAFFIN digests containing the accession sequences for cases in which they participated (due to high volume and time burden, trainees were often unable to manually keep track of accession numbers before PARAFFIN). Additionally, our institution fully digitized the anatomic pathology practice between the time first post-intervention survey and the second post-intervention survey trainees; scanned slides for a case are most easily accessed via the LIS. Another hypothesis is that trainees interpreted “looking up/retrieving final anatomic pathology reports” as “interacting with the LIS” during review of signed out cases in which they participated.

**Fig. 4:**
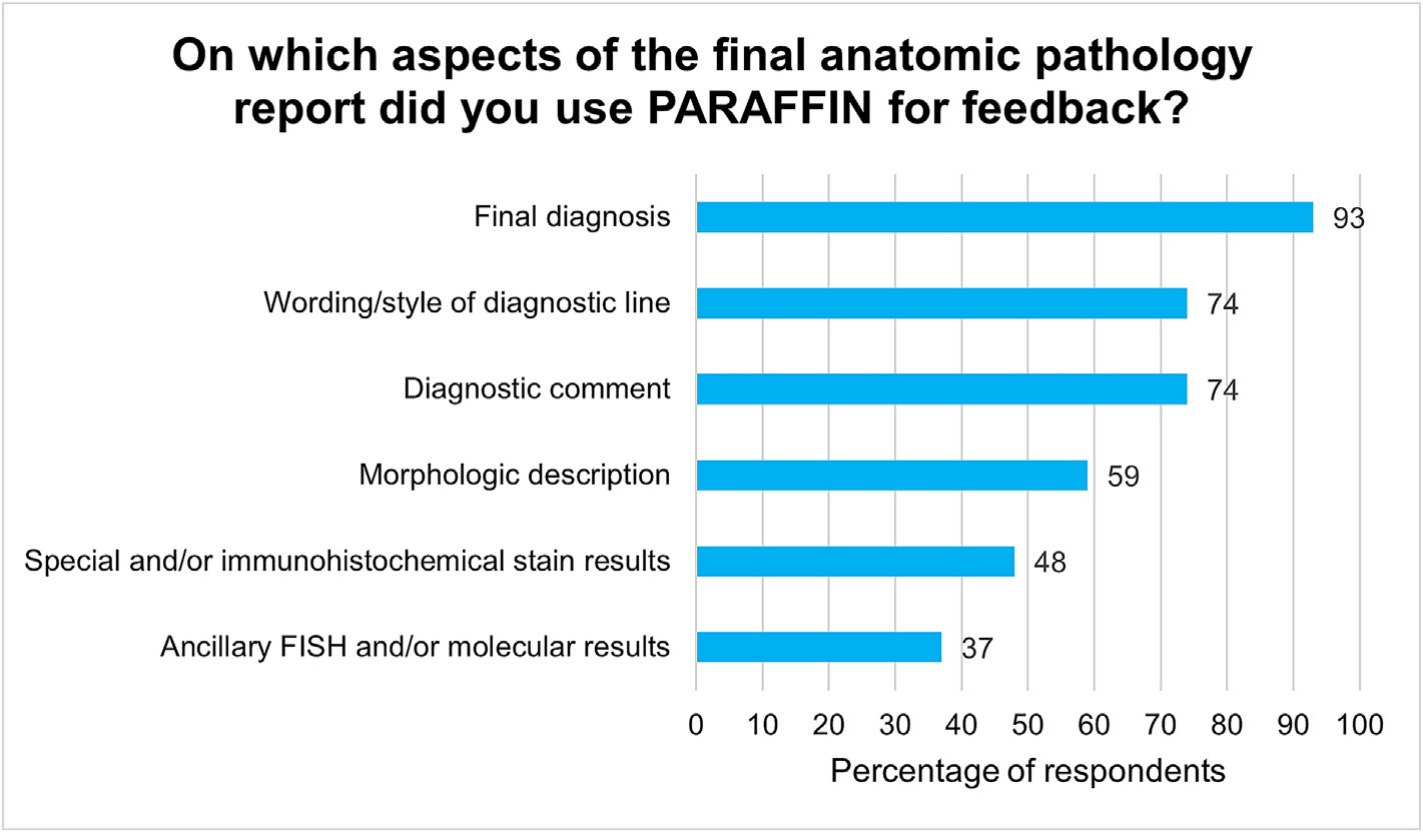
Survey results for the question “On which aspects of the final anatomic pathology report did you use PARAFFIN for feedback?” Results taken from the first follow-up survey (*n* = 27 respondents).

PARAFFIN's automation of the collection of final pathology reports provides significant utility to trainees by allowing them to spend less time on the manual, time-consuming process of looking up reports in the LIS. In the post-intervention surveys, 85% (23 of 27) and later 90% (18 of 20) of trainees reported that PARAFFIN allows them to spend more time reviewing the content of final anatomic pathology reports, rather than collecting reports. Importantly, trainees report using PARAFFIN digests for feedback on a number of aspects of pathology reporting (Fig. 4). Verification of the final diagnosis was most often used, followed by the wording/style of the diagnostic line and the diagnostic comment.

**Fig. 5:**
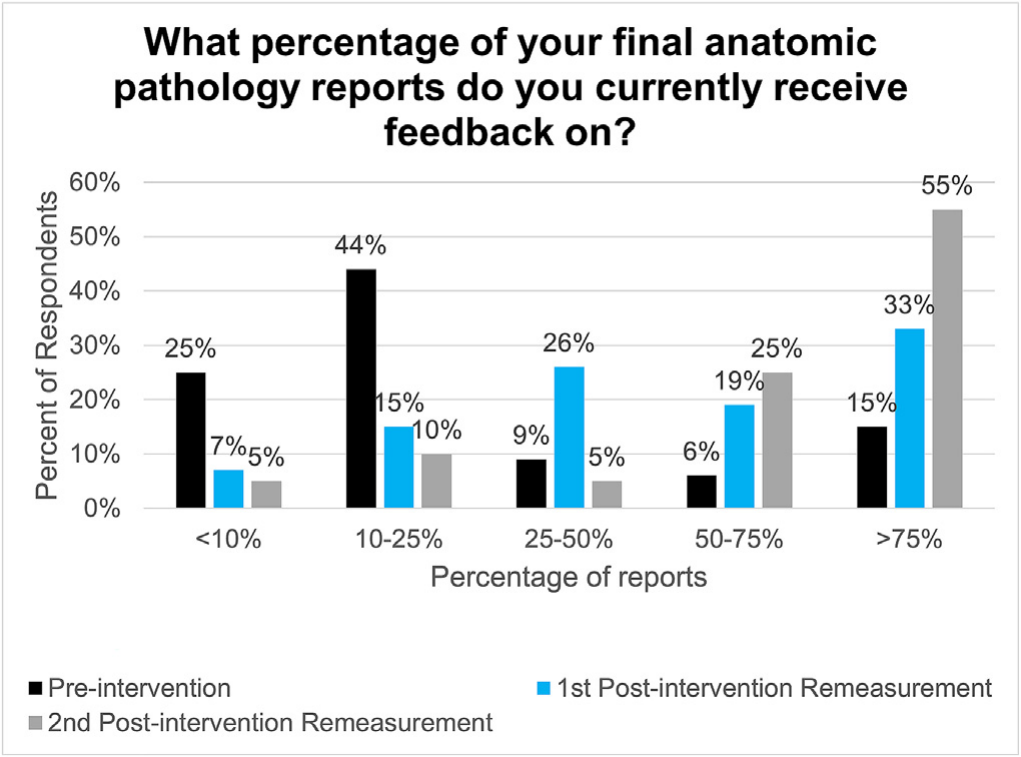
Survey results for the question “What percentage of your final anatomic pathology reports do you currently receive feedback on?” Answer options were “<10%”, 10–25%”, “25–50%”, “50–75%”, and “>75%”. (*n* = 32 respondents for the pre-intervention survey, *n* = 27 respondents for the first follow-up survey, and *n* = 20 respondents for the second follow-up survey).

Through automated receipt of their final anatomic pathology reports, PARAFFIN allows trainees to receive feedback on more of the cases in which they participated. In the pre-intervention survey, 16% (5 of 32) of trainees report receiving feedback on >75% of final anatomic pathology reports. Approximately 5 months after the implementation of PARAFFIN, this figure increased to 33% (9 of 27) and showed a sustained increase at our second post-intervention remeasurement 2 years later, with 55% of trainees (11 of 20) reporting receiving feedback on at least 75% of cases (Fig. 5). This represented a statistically significant improvement from baseline (*p*-value 0.007).

## Discussion

### Benefits of PARAFFIN

We propose that our implementation of PARAFFIN has a number of benefits to pathology trainees by removing barriers to their access of final pathology reports. PARAFFIN increases the effectiveness and sustainability of reviewing finalized cases by automating the time-consuming process of manually collecting reports. This decreases the amount of time trainees spend collecting reports (a clerical activity), allowing them to spend more time reviewing reports and cases (an educational activity).

By allowing learners to verify the final diagnosis of cases in which they participated, PARAFFIN increases diagnostic performance and captures lost learning opportunities by “closing the loop” on both routine and challenging cases, including those for which a trainee was unsure about their diagnosis. Crucially, PARAFFIN also provides trainees with the results of immunohistochemical and special stains and other ancillary testing, which may not have been initially available. Ideally, pathology attendings and trainees discuss the results of ancillary studies and how they inform both the final diagnosis and the report; however, temporal and spatial constraints (e.g., trainee has rotated to a different clinical service) mean that pathology staff may not always have the opportunity to have direct contact with the trainee for these discussions. While unable to completely replace this interaction, PARAFFIN bridges this potential gap, ensuring that these learning opportunities are not lost.

In addition to the verification of diagnoses and ancillary study results, providing trainees with final anatomic pathology reports aids in the development of a reporting vocabulary. In particular, trainees may benefit from reading the final diagnostic comment on challenging cases. Pathologists may include diagnostic comments in reports to convey nuance, provide differential diagnoses, explain additional important details, and express uncertainty when appropriate. These comments are considered an important part of pathology reporting,^11^ and as such, often require careful consideration and refinement, some of which may occur after the attending pathologist and trainee have met to discuss the case. Using final pathology reports of cases they have participated in as a model, trainees can develop their own reporting vocabulary.

Finally, PARAFFIN allows trainees to build and maintain a record of cases and reports that they have participated in, and can refer back to at any time throughout their training and beyond. Benefits of such a case log include the ability to look up a prior case to compare morphologic features and the ability to reference the wording of past reports. Receiving PARAFFIN digests over the entire course of residency training allows trainees to easily track their own coursework for the purpose of American Board of Pathology case logging. A case log also has significant utility in both education and research. For example, trainees are often asked to present interesting cases at didactic conferences; having a record of all of their cases significantly cuts down on the time needed to search for a specific case or diagnosis.

In addition to its educational value for trainees, staff pathologists receiving PARAFFIN digests have noted its utility in providing them with feedback on cases for which they were consulted, as well as enabling them to reference the wording of previous reports.

### Limitations

Our implementation of PARAFFIN has several limitations. First and foremost, any automated feedback mechanism, including PARAFFIN, is not an alternative for double scoping or in-person feedback provided by an attending pathologist to trainees. Rather, our intent is for PARAFFIN to be a supplemental feedback mechanism for residents and fellows, allowing learners to verify their diagnosis, understand pathology reporting criteria and wording, and build a record of cases in which they have participated.

Another limitation is that results of ancillary studies that are pending at the time of the final pathology report (and are signed out by the attending pathologist as addenda) are not included in PARAFFIN digests, as these addenda are not stored in the Tableau workbook from which PARAFFIN derives its data.

A notable limitation is that our implementation of PARAFFIN does not provide trainees with prior versions of the anatomic pathology reports (such as the trainee's “final” report before it was edited by the attending pathologist). Certain LIS implementations, such as Epic Beaker, allow for storage of a “resident diagnosis”; however, our institution does not make use of such a field in our reporting. Because of these limitations of our system, PARAFFIN does not show tracked changes to final anatomic pathology reports. Other implementations of case feedback systems for trainees^12^ make use of an “agreement score” (e.g. “agree,” “partially agree,” or “disagree”) provided by attending pathologists to rate a trainee's diagnosis and report; our institution does not currently utilize such a scoring system. Utilizing natural language processing to compare the trainee and attending diagnoses, calculate the degree of similarity, and report this as a single statistic^13^ could be a useful addition to our implementation; however, the current reporting standards within our institution and the limitations of our LIS preclude the addition of such a feature.

### Future directions

Given the widespread adoption of digital pathology, including at our own institution, future implementations of PARAFFIN will link to digital slides for each case, allowing trainees to easily re-review not only the final pathology report, but also the histopathology. Such a feature would be especially useful in those cases in which an attending pathologist disagreed with a trainee on the final diagnosis, allowing the trainee to re-review the slides to confirm their understanding.

## Conclusions

Feedback is essential to the training of pathology residents and fellows, but given time constraints and competing obligations, can be difficult to provide in a timely manner. Our solution is an automated case feedback system, PARAFFIN, written in R and utilizing a Posit Connect server, which sends trainees a weekly digest of all the final pathology reports for cases in which they have participated. While PARAFFIN is not a substitute for in-person feedback, our implementation supplements resident and fellow education by ensuring pathology trainees are consistently informed of the final outcome of cases in which they have been involved. PARAFFIN automates the time-consuming process of manually collecting reports, addressing the limited amount of time trainees have and allowing learners to focus on reviewing reports, rather than retrieving them. We believe that automated case feedback tools such as PARAFFIN benefit pathology trainee education at multiple levels.

## Funding

This research did not receive any specific grant from funding agencies in the public, commercial, or not-for-profit sectors.

## Declaration of competing interest

The authors declare that they have no known competing financial interests or personal relationships that could have appeared to influence the work reported in this article.

## Appendix A Supplementary data

Baseline survey, which was distributed to all 43 pathology trainees at our institution, prior to the implementation of PARAFFIN. Study data were collected and managed using REDCap. Supplementary material 1

Follow-up survey, which was distributed to all 32 pathology trainees at our institution who participated in the baseline survey, approximately 5 months after the implementation of PARAFFIN. The follow-up survey was re-distributed 2 years after the implementation of PARAFFIN to conduct second, long-term re-measurement of PARAFFIN's impact. Study data were collected and managed using REDCap. Supplementary material 2
