# Supplementary material for: PARAFFIN: A software tool for Pathology Report Automated Feedback for Improved Education of anatomic pathology trainees
Source: J Pathol Inform. 2025 Feb 13;17:100424. doi: 10.1016/j.jpi.2025.100424 (PMC11938144; doi:10.1016/j.jpi.2025.100424)
Supplement: Supplementary file 1 — Baseline survey, which was distributed to all 43 pathology trainees at our institution, prior to the implementation of PARAFFIN. Study data were collected and managed using REDCap. [file mmc1.pdf]

PARAFFIN Baseline

Record ID

____

Current level of pathology training (PGY)

- ◯ PGY-1
- ◯ PGY-2
- ◯ PGY-3
- ◯ PGY-4
- ◯ PGY-5
- ◯ PGY-6
- ◯ PGY-7
- ◯ PGY-8

Current pathology role

- ◯ Pathology resident
- ◯ Pathology fellow

Anatomic Pathology Report Training / Resources

Have you received formal training to draft final anatomic pathology reports?

- ◯ Yes
- ◯ No

What resources do you use when drafting final anatomic pathology reports (see all that apply)?

- ☐ Log of past final anatomic pathology reports
- ☐ Libre Pathology
- ☐ Pathology Outlines
- ☐ Other pathology website
- ☐ Other resource

What other pathology website(s) do you use when drafting final anatomic pathology reports?

____

What other resource(s) do you use when drafting final anatomic pathology reports?

____

Anatomic Pathology Report Feedback

How have you received (or collected) feedback on your final anatomic pathology reports?

- ☐ None
- ☐ Verbal feedback from the consultant
- ☐ Manual audit in SoftPathDx
- ☐ Review signed out case shelf
- ☐ Other

What other resource(s) have you used to receive (collect) feedback on you final anatomic pathology reports?

____

What percentage of your final anatomic pathology reports do you currently receive feedback on?

- ◯ < 10%
- ◯ 10-25%
- ◯ 25-50%
- ◯ 50-75%
- ◯ >75%

For those final anatomic pathology reports that you do receive feedback on, what percentage of that feedback is related to:

	< 10%	10-25%	25-50%	50-75%	>75%
Final diagnosis	◯	◯	◯	◯	◯
Final diagnostic line (wording/style)	◯	◯	◯	◯	◯
Diagnostic comment	◯	◯	◯	◯	◯
Morphologic description	◯	◯	◯	◯	◯
Special and/or immunohistochemical stain reporting	◯	◯	◯	◯	◯
Ancillary FISH and/or molecular result reporting	◯	◯	◯	◯	◯

Over the course of an average week, how much time per day do you spend:

	None	< 30 minutes	30 minutes to 1 hour	>1 hour
Looking up / retrieving final anatomic pathology reports?	◯	◯	◯	◯
Reviewing final anatomic pathology reports?	◯	◯	◯	◯
Creating / maintaining a log of final anatomic pathology reports?	◯	◯	◯	◯

Why do you not spend time looking up / retrieving final anatomic pathology reports?

- ☐ Too time-consuming / not enough time
- ☐ Not beneficial to education
- ☐ Don't care to
- ☐ Other

What other reason(s) do you not spend time looking up / retrieving final anatomic pathology reports?

____

Why do you not create or maintain a log of final anatomic pathology reports?

- ☐ Too time-consuming / not enough time
- ☐ Not beneficial to education
- ☐ Don't care to
- ☐ Other

What other reason(s) do you not spend time creating or maintaining a log of final anatomic pathology reports?

____

Does the time it takes to retrieve final anatomic pathology reports hinder/prevent you from reviewing such reports while the cases are still fresh in your mind?

- ◯ Yes
- ◯ No

Would a log of final anatomic pathology reports you have participated in be valuable to your pathology education?

- ◯ Yes
- ◯ No

How would you use a log of the final anatomic pathology reports you participated in?	☐ Feedback on how to improve final anatomic pathology reports<br>☐ Reference for preparing future final anatomic pathology reports<br>☐ Create summary of cases seen for board certification or job applications<br>☐ Other
In what other way(s) would you use a log of final anatomic pathology reports you participated in?	__________________________________
