# Supplementary material for: PARAFFIN: A software tool for Pathology Report Automated Feedback for Improved Education of anatomic pathology trainees
Source: J Pathol Inform. 2025 Feb 13;17:100424. doi: 10.1016/j.jpi.2025.100424 (PMC11938144; doi:10.1016/j.jpi.2025.100424)
Supplement: Supplementary file 2 — Follow-up survey, which was distributed to all 32 pathology trainees at our institution who participated in the baseline survey, approximately 5 months after the implementation of PARAFFIN. The follow-up survey was re-distributed 2 years after the implementation of PARAFFIN to conduct second, long-term re-measurement of PARAFFIN's impact. Study data were collected and managed using REDCap. [file mmc2.pdf]

PARAFFIN Follow-Up

Record ID

____

Current level of pathology training (PGY)

- ◯ PGY-1
- ◯ PGY-2
- ◯ PGY-3
- ◯ PGY-4
- ◯ PGY-5
- ◯ PGY-6
- ◯ PGY-7
- ◯ PGY-8

Current pathology role

- ◯ Pathology resident
- ◯ Pathology fellow

Final Anatomic Pathology Report Email Digest

Did you receive a weekly email containing a digest of recent final anatomic pathology reports that you participated in?

- ◯ Yes
- ◯ No

What percentage of weeks did you open and review this email digest of recent final anatomic pathology reports?

- ◯ < 10%
- ◯ 10-25%
- ◯ 25-50%
- ◯ 50-75%
- ◯ >75%

Anatomic Pathology Report Feedback

How have you received (or collected) feedback on your final anatomic pathology reports?

- ☐ None
- ☐ Verbal feedback from the consultant
- ☐ Manual audit in SoftPathDx
- ☐ Review signed out case shelf
- ☐ Email digest of final anatomic pathology reports
- ☐ Other

What other resource(s) have you used to receive (collect) feedback on you final anatomic pathology reports?

____

What percentage of your final anatomic pathology reports do you currently receive feedback on?

- ◯ < 10%
- ◯ 10-25%
- ◯ 25-50%
- ◯ 50-75%
- ◯ >75%

For those final anatomic pathology reports that you do receive feedback on, what percentage of that feedback is related to:

	< 10%	10-25%	25-50%	50-75%	>75%
Final diagnosis	○	○	○	○	○
Final diagnostic line (wording/style)	○	○	○	○	○
Diagnostic comment	○	○	○	○	○
Morphologic description	○	○	○	○	○
Special and/or immunohistochemical stain reporting	○	○	○	○	○
Ancillary FISH and/or molecular result reporting	○	○	○	○	○

Over the course of an average week, how much time per day do you spend:

	None	< 30 minutes	30 minutes to 1 hour	>1 hour
Looking up / retrieving final anatomic pathology reports?	○	○	○	○
Reviewing final anatomic pathology reports?	○	○	○	○
Creating / maintaining a log of final anatomic pathology reports?	○	○	○	○

Why do you not spend time looking up / retrieving final anatomic pathology reports?

- ☐ Too time-consuming / not enough time
- ☐ Not beneficial to education
- ☐ Don't care to
- ☐ Use of email digest of final anatomic pathology reports (no time required)
- ☐ Other

What other reason(s) do you not spend time looking up / retrieving final anatomic pathology reports?

____

Why do you not create or maintain a log of final anatomic pathology reports?

- ☐ Too time-consuming / not enough time
- ☐ Not beneficial to education
- ☐ Don't care to
- ☐ Use of email digest of final anatomic pathology reports (no time required)
- ☐ Other

What other reason(s) do you not spend time creating or maintaining a log of final anatomic pathology reports?

____

Final Anatomic Pathology Report Email Digest

How satisfied were you with the email digest of final anatomic pathology reports you participated in as a feedback mechanism?

- ◯ Not satisfied at all
- ◯ A little satisfied
- ◯ Moderately satisfied
- ◯ Very satisfied
- ◯ Extremely satisfied

Which aspects of the final anatomic pathology report did you use the weekly email digest for feedback?

- ☐ Final diagnosis
- ☐ Final diagnostic line (wording/style)
- ☐ Diagnostic comment
- ☐ Morphologic description
- ☐ Special and/or immunohistochemical stain reporting
- ☐ Ancillary FISH and/or molecular result reporting

What improvements would you suggest for the email digest of final anatomic pathology reports you participated in?

____
